# The chemosensory system of the *Drosophila* larva: an overview of current understanding

**DOI:** 10.1080/19336934.2021.1953364

**Published:** 2021-10-06

**Authors:** Nikita Komarov, Simon G. Sprecher

**Affiliations:** Institute of Cell and Developmental Biology, Department of Biology, University of Fribourg, Fribourg, Switzerland

**Keywords:** *Drosophila* larva, chemosensation, neural circuits, processing, behaviour, model organisms

## Abstract

Animals must sense their surroundings and be able to distinguish between relevant and irrelevant cues. An enticing area of research aims to uncover the mechanisms by which animals respond to chemical signals that constitute critical sensory input. In this review, we describe the principles of a model chemosensory system: the *Drosophila* larva. While distinct in many ways, larval behaviour is reminiscent of the dogmatic goals of life: to reach a stage of reproductive potential. It takes into account a number of distinct and identifiable parameters to ultimately provoke or modulate appropriate behavioural output. In this light, we describe current knowledge of chemosensory anatomy, genetic components, and the processing logic of chemical cues. We outline recent advancements and summarize the hypothesized neural circuits of sensory systems. Furthermore, we note yet-unanswered questions to create a basis for further investigation of molecular and systemic mechanisms of chemosensation in *Drosophila* and beyond.

## Introduction

Animal central nervous systems (CNS) are responsible for integrating environmental information to determine an optimal behavioural output. Each decision must be directly linked to the canonical, albeit simplistic, rule of Darwinian evolution: improving an organism’s probability of feeding, fighting, fleeing, and, ultimately, mating to pass on its genetic information to offspring. While nervous systems are as diverse as the behavioural strategies they create, key principles of CNS organization and integration remain constant across phylogeny [1]. Understanding molecular and physiological features of brains, such as that of humans and other vertebrates, is key in elucidating fundamental principles behind health and disease. However, the complexity of a mammalian brain [2] hinders our ability to understand how individual neurons and synapses integrate into the vaster tapestry of processing connections, which result in the limitless behaviours mammals exhibit. Instead, by studying simpler nervous systems, such as that of the fruit fly *Drosophila melanogaster*, it is possible to uncover fundamental mechanisms of nervous systems and relate these findings to more applicable questions. Moreover, the larva of the fly presents us with a numerically simpler model for investigating the function and organization of nervous systems. Due to this, and the vast genetic toolkit allowing for manipulation of the nervous system down to a single-cell level, the *Drosophila* larva has become a compelling model of studying the nervous system [3–9]. However, despite the relative simplicity of this system, much remains to be learned about the fundamental principles of neural circuits at this level.

One tangible aspect of this model is its avoidance and attraction behaviour in relation to environmental cues. The ability to locate and identify ecologically relevant compounds is crucial in an animal’s ability to fulfill its evolutionary tasks, and more so when the environment experienced by the animal is chemically rich. In this review, we focus on principles of chemosensation and the mechanisms of neuronal circuits that result in key behavioural outputs.

## Molecular basis of chemosensation

The first point of interaction between the larva and its environment is peripheral sensory neurons, which express different families of chemoreceptor genes. The most studied of these are the Odorant Receptor (OR) family [10–12], Ionotropic Receptor (IR) family [13–15], and the Gustatory Receptor (GR) family [16–19]. Non-canonical receptors, such as the transient receptor potential (TRP) channels, have been conclusively implicated in taste [16,19–23], while pickpocket (Ppk) sodium channels have been shown as key to salt sensation, including the ability to segregate salt concentrations resulting in varied behavioural outputs [24–26].

All identified neuronal chemoreceptor proteins expressed in the larva are believed to form ion channels that result in the depolarization of chemosensory neurons through ligand-mediated channel opening [14,26,27]. It must be noted, however, that the underlying mechanisms of interaction between ligand and receptor, as well as channel opening, have not been definitively uncovered. Despite this, peripheral chemosensation in *Drosophila* is thought to be distinct from that of vertebrates because of the apparent lack of G-protein-coupled receptor (GPCRs) involvement at the olfactory sensory neuron (OSN) membrane [12,27]. This contrast is disputed, as some have suggested that a metabotropic component is present, while others have yet been unable to link G-proteins to olfactory function, with a notable exception for CO_2_ sensing neurons [28–31]. Since CO_2_ sensation occurs via the gustatory receptor family, it is possible that an interaction between the G-protein G_aq_ and the GR proteins exists. However, such studies were performed in the adult, and no evidence exists to support or dispute the role of GPCRs in larval chemosensation.

ORs are a diverse gene family that code for 7-transmembrane helix monomers, which in turn assemble into hetero-tetramers consisting of at least one obligatory co-receptor subunit (Olfactory Receptor Co-receptor, Orco), and, in the case of the larva, one of the 21 tuning OR genes [10–12,32]. Similarly, IR genes also code for transmembrane monomers, and are also believed to form hetero-tetramers, with at least one obligatory co-receptor (Ir25a, Ir76b, or Ir84a) and tuning IR subunits [13–15,33]. Beyond this, similarities between ORs and IRs are sparse. The OR family is unique to insects and responds to a wide variety of volatile compounds and pheromones [15,34], while the IRs in the larva are implicated in taste, thermosensation and hygrosensation. IRs are believed to have evolved from the ionotropic glutamate receptor (iGluR) gene family which persists throughout the animal kingdom and is present at the post-synaptic membrane to facilitate glutamate synapse transmission [27,33]. The Ppk gene family also includes subunits homologous to the epithelial sodium channel (ENaC), which is thought to remain constitutively open, thus causing depolarization in the presence of a high concentration of cations [35]. This allows for the channel’s function as a salt receptor [25]. Another member of this family, Ppk25, has been shown to amplify odorant responses in neurons where it is expressed [36]. Finally, the TRP channel family also forms transmembrane tetramers that, depending on the extracellular component, can act as both ligand-gated, as in the case of bitter sensation, or non-ligand gated channels, such as in the case of thermosensation [20,21,37]

The GR family is distantly related to the OR family; however, these receptors in general sense non-volatile compounds, implicating them in taste function [18,19,38]. No GR co-receptor has been identified, and the functional organization of these genes remains to be elucidated [39]. An exception to the properties of their relatives are the receptors Gr21a and Gr63a, which serve a pseudo-olfactory function through their specific role in CO_2_ sensation [40–43], a function conserved across insect families [42].

Chemosensory receptors are expressed mainly in the dendrites of peripheral neurons; however, some expression is evident in the CNS, and is believed to be relevant for monitoring the internal state [38,44,45].

## Anatomical overview

**Figure 1.**
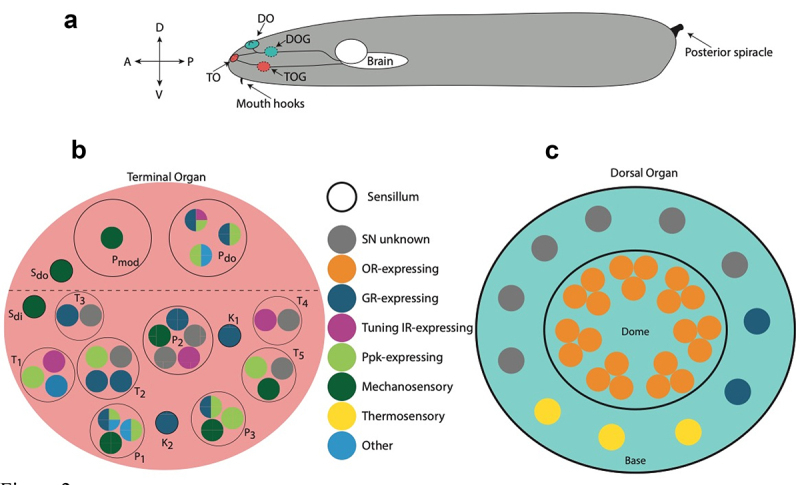
**A**: Broad organization of primary external larval chemosensory organs in context. **B**: The terminal organ (TO, red) contains dendrites of neurons that express a wider variety of chemosensory genes. The organizational logic of sensilla and dendrites has not been uncovered. Sensilla are named according to the morphology as described by Rist and Thum., 2017: Papillum (P1-3, dorso-lateral (do), modified (mod)); Pit (K1-5); Spot dorso-lateral (do), and distal (di); Knob (K1, K2). **C**: The dorsal organ (DO, blue) contains 2 sensilla (Dome, Base). The Dome contains mainly OR-expressing neurons arranged in triplets, implicating it in olfactory function. The identity of most Base neurons remains unknown. Furthermore, presence of some IRs has been shown in the DO, however the identity of the neurons expressing them is unknown [55], thus they have not been included. Partially adapted from Apostolopolou et al., 2015, and Rist and Thum, 2017

In comparison with the adult, the larval external chemosensory system is much simpler, consisting of three peripheral sensilla: the dorsal organ (DO), terminal organ (TO) and the ventral organ (VO); in addition to three pharyngeal sensilla: the dorsal (DPS), ventral (VPS) and posterior (PPS) sensory organs [46]. Other ganglia, such as the dorsal pharyngeal organ (DPO), have been suggested, but not adequately characterized [46]. Each sensillum has an associated ganglion that houses sensory neurons, which extend dendrites into the external organs [4,47–49]. Classically, it was thought that each organ is responsible for one type of sense; however, it has been shown that the roles overlap [50]. The DO and TO are the main and most-characterized organs (Figure 1).

## Basic logic of olfactory neural pathways

The DO and its associated ganglion DOG are the primary olfactory organ, a result of the dendrites of 21 OSNs resident in the dome of the DO, arranged in triplets [47,51–53] (Figure 1), in addition to 11 largely uncharacterized OSNs in the periphery (base) of the DO, some of which are believed to be involved in mechanosensation and thermosensation due to the expression of relevant receptor genes [54]. The remaining neurons are uncharacterized, but are not believed to be OR-expressing. While some other genes have been shown to be present, such as some IRs, they have not yet been mapped to individual neurons [55]. The dome and base of the DO form identifiable sensilla. OSNs project from the DOG to the antennal lobes (AL), with each OSN projecting onto a unique glomerulus within the AL via the antennal nerve [46,56–59]. Projections of peripheral neurons are yet to be uncovered. Each larval OSN expresses a tuning OR that characterizes the neuron, in a way that is logically consistent with even the vertebrate system [11,13–15,33,60–64]. While Orco is known to be co-expressed in all OR OSNs, rare co-expression of tuning ORs has also been described, but not deeply explored [65]. Furthermore, the DO has been implicated in taste sensation [50]; however, the molecular underpinnings of this mechanism have not yet been investigated. OSNs are assisted by Odorant Binding Proteins (OBPs) that, while largely uncharacterized, are believed to facilitate the transport of hydrophobic odour compounds through the aqueous haemolymph that surrounds olfactory dendrites [66–68].

**Figure 2.**
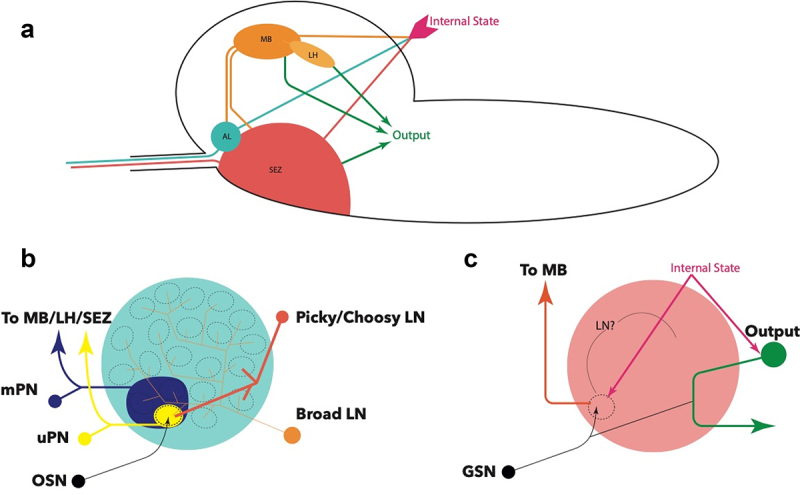
**A**: Topological organization of the principal processing neuropils in the brain. AL -antennal lobe; SEZ – suboesophageal zone; MB – mushroom body; LH – lateral horn of the mushroom body. **B**: Glomerular organization of the antennal lobe, with example LNs responsible for modulating and moderating signals from OSNs. mPNs convey inhibitory signals to other brain centres, while uPNs convey excitatory signals. The nature of mPN neurotransmitters is not known. **C**: Current understanding of SEZ organization, showing both monosynaptic and polysynaptic pathways of gustatory sensory neurons (GSN), and the influence of internal state on these pathways. The presence and identity of local neurons has not been elucidated, but has been proposed

**Figure 3.**
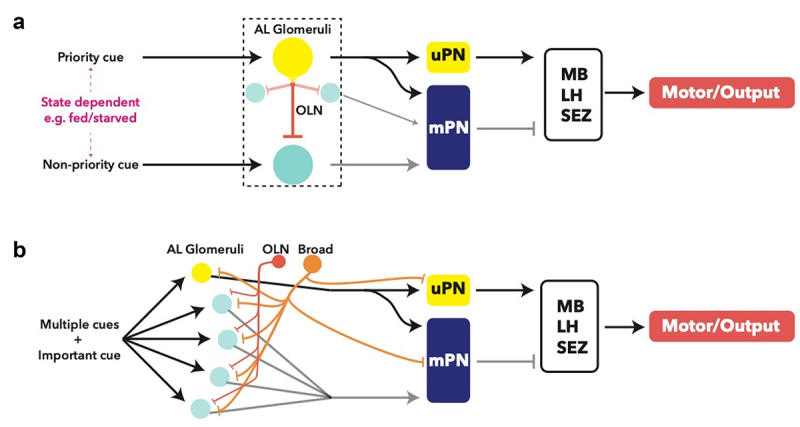
Processing logic within the glomerular system of the antennal lobe. **A**: putative ‘priority’ and ‘non-priority’ cues are determined by the identity of the glomerulus and the internal state, for example fed vs. starved, which results in varied modulation by local neurons such as Picky, downstream of OSNs [72]. Oligoglomerular local neurons (OLN) are inhibitory to other glomeruli when activated, modulating the output of the active glomerulus to other brain centres via a uPN for processing. **B**: proposed principle of processing of multiple cues, only one of which is an ‘important’ cue based on connectomic studies. The active glomerulus (yellow) will activate an OLN resulting in stronger inhibition of other glomeruli (blue), modulating their output to other brain centres via mPNs

OSNs project from the DO via the antennal nerve (AN), where each neuron maps to a distinct, non-overlapping glomerulus (Figure 2) [69,70]. Antennal lobe glomeruli are the first neuropils in the olfactory processing circuitry [58,70]. The glomeruli are wired with networks of mainly inhibitory, GABAergic local neurons (LN) that allow for signal modulation and integration. A notable exception is that of Picky LNs, which are chiefly glutamatergic [71]. Furthermore, a recent study has functionally characterized the role of a Picky LN in state-dependent modulation of behaviour, shedding light on the processing role of these neurons [72]. Connectomic studies have described different varieties of LNs present in each lobe. Broad LNs connect to all neurons in the AL, and modulate lobe-wide activity through suppression of outgoing information by inhibition of the integrating uniglomerular and multiglomerular projection neurons (uPNs and mPNs, respectively) (Figure 3b) [70]. This allows incoming information from different OSNs to form a robust output to higher brain centres by masking background activity in ‘noisy’ environments while modulating the dynamic range of each odorant response (Figure 3) [70,71,73–75]. Other LNs, including Picky and Choosy, act on behalf of specific and distinct glomeruli [70]. They appear to strongly mediate aversive and attractive behaviours by contextualizing (i.e. highlighting) the incoming signal through inhibition of co-lateral glomeruli and other LNs, forming a hierarchical structure (Figure 3) [58,70,76,77]; however, their full functions are still being elucidated.

A key feature of animal olfactory systems is the presence of neuronal habituation, whereby repeated exposure to a stimulus results in a dampened response, especially in the absence of conditioning stimuli [78]. Habituation is typically multifactorial; however, both adult and larval mutant studies have shown that the *Drosophila* mechanism revolves around metabotropic depolarization coincidence detection. Here, repeated stimulation of LNs by OSNs causes increased cAMP levels, which increases neurotransmitter release (in this case, inhibitory GABAergic transmission). Thus, LNs mediate habituation by increasing their inhibitory activity upon repeated stimulation by an OSN [79–81].

Connectomic studies have suggested that projection neurons aggregate information from the AL and converge into the Antennal Lobe Tracts (ALT) [70]. The medial ALT (mALT) projects from the antennal lobe to the mushroom body (MB) calyx, and contains all uPNs with the exception of Or35a, which projects to the lateral horn (LH) via the lateral ALT (lALT). This story is similar to the mPNs, which are mostly projected via mALT with the exception of cobra mPN and seahorse mPN [70]. The mushroom body is the main centre for olfactory memory formation due to the local dopaminergic and octopaminergic neuron processing that affects MB input neurons (MBIN), MB output neurons (MBON), and Kenyon cells (KC) [82–86]. These principles are supported by the similar findings from the adult [74,87,88]. A key question for current investigation is whether such processing is bilateral or unilateral, i.e. whether unilateral stimulus is sufficient for a behavioural response. While there is ample bilateral interconnectedness between the MBs, including via MBONs [82], functional validation of bilateral processing is proving to be an experimental challenge. mALT PNs continue to the lateral horn for processing of a behavioural response, which can be attractive (i.e. ‘seek’) through positive chemotaxis, or aversive (i.e. ‘flee’) through turning and negative chemotaxis. As the name suggests, mPNs integrate the processed outputs from multiple glomeruli, in both an inhibitory and excitatory fashion in the adult [71] and in an unclear manner in the larva, and project to the MB and LH, as well as the suboesophageal zone (SEZ), which is the principle taste processing neuropil [58,70,89,90].

Despite the numerical simplicity of the larval olfactory system, it is able to respond to and discriminate from a broad range of odours. This makes this system an exciting model for investigating complex neuronal coding from a simple input system. For example, the *Drosophila* olfactory system model is used to investigate development [91–93], evolution [94–97], and novel genetic mechanisms [98] underpinning the function of neural circuits. Furthermore, findings from larval olfactory systems have been transferred to uncover principles further afield, such as in vector control biology [99–103].

## Taste organs and sensory neurons

Compared with olfaction, underpinnings of the gustatory network of the *Drosophila* larva are much less understood. One reason for this is the relative complexity of taste compared with olfaction. For instance, as described above, a typical OSN expresses a single tuning receptor gene, which maps to a distinct glomerulus in the AL in a non-overlapping manner. By comparison, the primary receptor genes of the taste system, the GR family, are co-expressed in a currently unexplored manner in gustatory receptor neurons (GRNs) resulting in a degree of multimodality, while peripheral neurons map into overlapping areas of the SEZ (Figures 1,2) [18,38,104,105]. As mentioned, IRs, TRPs and PPK channels have also been characterized in taste circuits due to their expression in larval GRNs (Figure 1) and their known responses to a wide range of stimuli, both chemosensory and environmental [23,33,54,106,107]. Furthermore, multiple-taste organs exist, such as the external Terminal Organ (TO, primary taste organ) and Ventral Organ (VO), and a number of internal pharyngeal and enteric sensory regions [106,108,109]. The complexity of the taste system creates an interesting evolutionary conundrum, since each *Drosophila* larva hatches, feeds, and undergoes pupal metamorphosis confined to the rotting fruit on which it was laid, spending most of the larval stage buried and continuously feeding in the flesh of the fruit [8]. At most, the larva must find patches of fruit at the optimum stage of fermentation. Thus, a complex gustatory system appears redundant when viewed in this context. Understanding the principles of gustatory function may therefore uncover novel behavioural and evolutionary strategies underlying feeding decisions and sensorimotor circuits [3,90].

The larva exhibits a narrow, well-characterized array of behaviours in response to tastants. It is aversive to bitter compounds, and attracted to sugars, yeast, and certain amino-acids, including in a combinatorial discriminatory manner, as well as with concentration-dependent processing [110–115]. Interestingly, the mechanisms of sugar sensing, arguably one of the most important chemical cues, is not entirely understood. Specifically, no external sugar-sensing mechanism has yet been characterized. Gr43a is the canonical sugar receptor; however, it is only expressed in internal organs and some parts of the brain [44]. In fact, central neurons expressing Gr43a are sufficient for sugar sensation by themselves, likely by sensing the sugar concentration in the haemolymph and bypassing the SEZ altogether. In this way, sugar acts as both a peripheral (external) stimulus, as well as an internal metabolic state stimulus [44]. Indeed, Gr43a is known to be widely expressed, yet specifically tuned to sugar. In the adult *Drosophila*, for instance, Gr43a is expressed in the taste organs for peripheral sugar sensing, in the brain as a metabolic signal similar to the larva, in the gut to modulate gastrointestinal activity, and even in the uterus to control post-mating behaviours in response to fructose present in the seminal fluid [116]. This reiterates the necessity for sugar sensing, due to it being an essential cue for energy availability.

## Taste circuits

Recently, neural circuits underlying taste processing have begun to be uncovered. The TO houses dendrites of 32 neurons from the Terminal Organ Ganglion (TOG), in addition to dendrites of three sensory neurons from the Dorsal Organ Ganglion (DOG) [108]. Of these, 10 are GR expressing taste neurons, and are classified by anatomical position rather than by tuning receptor expression. Four neurons express IRs, and an equal number express Ppk channels. One neuron expresses the *serrano* protein, which is known to be necessary for high-salt avoidance when coupled with Ppk19 [24], while another neuron expresses an uncharacterized protein related to the MB memory circuit [106,117]. Dendrites uniquely project to one of 14 sensilla of 5 types and each sensillum contains between 1 and 4 GSNs [106]. Notably, the C6 neuron is specifically tuned to CO_2_, and thus shows similarities to olfactory receptor neurons [118]. The role of the remaining neurons remains to be fully uncovered; however, they have been attributed to thermo and hygrosensation, pheromone sensation and mechanosensation (Figure 1) [54,108,110]. The organizational logic of TO sensilla and dendrites is unclear, and needs further investigation.

**Figure 4.**
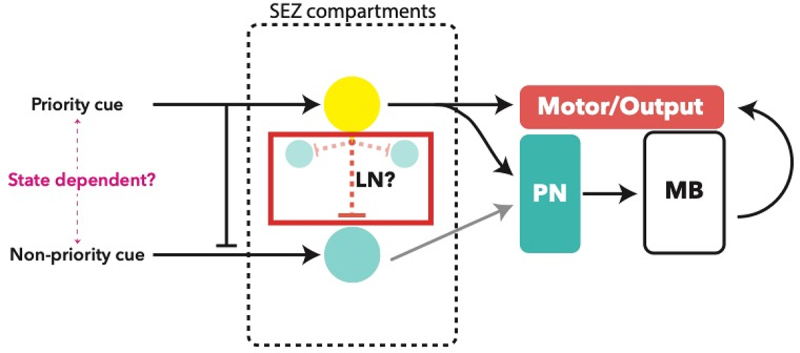
Proposed processing logic of a taste circuit. Internal state may lead to prioritization of one cue over another. Axo-axonal inhibition may facilitate lateral inhibition in a manner similar to local neurons (LN) which have not been described in the SEZ. Outputs from an SE compartment may be monosynaptic, *i.e*. directly resulting in a motor output, or polysynaptic, involving processing and signal integration by the mushroom body (MB) via projection neurons

The role of most TOG neurons remains uncertain [54]. Taste information is carried from external organs to the CNS via three pharyngeal nerves: antennal nerve (AN), maxillary nerve (MxN), and protothoracic accessory nerve (PaN). Afferent nerve bundles converge onto distinct and non-overlapping synaptic compartments of the SEZ [105]. A notable point here is the continuous development of the SEZ throughout the embryonic and larval stages [89], which must be considered when outlining the anatomical and connective organization. Such studies have looked at the 1^st^ instar (L1) larval stage; thus, it is not known whether this organization is conserved in later larval stages. Unlike the wiring of the olfactory circuit, much of the signal processing is suggested to occur through axo-axonal connections of the sensory neurons, albeit the nature of these connections, such as the neurotransmitter identity, is not known [105]. Local processing appears to also be abundant, although identities of local neurons are not characterized (Figure 4) [3,90,105]. Additionally, the polysynaptic circuits strongly resemble the processing of olfactory information, with efferent SEZ neurons projecting to the LH via the MB calyx [105]. This may be the main taste-learning mechanism in the larva, but this is yet to be elucidated, as well as the odour-taste learning pathway, whereby an odour is linked to an appetitive tastant and memory formation is believed to predictably occur via dopaminergic input to the MB [85,86].

These levels of processing may explain the behavioural inhibition observed when attractants (e.g. sweet) are coupled with aversive compounds (e.g. bitter) [110,111]. Furthermore, connectomic analysis has shown that almost half of the motor output signals are generated from a monosynaptic connection between internal sensory (enteric and pharyngeal) and output neurons (neuroendocrine, pharyngeal motor), indicating a prevalence of direct behavioural responses to external and internal stimuli such as the metabolic state [105,119]. While functional validation of taste circuits is scarce, connectomic analysis predicts that distinct areas of the SEZ are responsible for integrating external signals into a specific output; such as the neuroendocrine or motor neurons. Predictably, the SEZ output neurons are a key target for MBONs, adding an additional layer of decision-making. Here, the monosynaptic circuits directly affect the necessary motor outputs, such as the feeding behaviour (pharyngeal pumping) output for an appetitive tastant sensory input. In other cases, a larger variety of parameters are consideredfor example, the presence of additional tastants or odorants in the headspace, allowing learned responses from the MB to affect behavioural output via polysynaptic circuits involving MBONs [90,105,119].

However, it is also evident that much processing occurs at the level and downstream of the SEZ in higher brain centres. For example, the integration of olfactory and gustatory information for decision-making and learning in the MB via polysynaptic feeding circuits integrates most of the peripheral inputs [17,85,90,120–122]. How this higher signal processing modulates behavioural outputs remains unclear; however, evidence suggests that the integration of olfaction, taste, and other modalities in the ALs and MBs may be more crucial than classically thought, including reciprocal connections between the AL and SEZ [85,121,123,124]. This is further supported by the interconnectedness of Kenyon cells, fundamental neurons of the mushroom body, which receive inputs from projection neurons of all primary sensory neuropils [86]. Additionally, one must consider the role of neuroendocrine cells, such as the *Drosophila* neuromedinU homolog (Hugin). Hugin, and its vertebrate counterpart, act to modulate feeding and locomotive behaviour through a peptidergic neuromodulatory effect, rather than through direct synaptic relays [125–127]. Another hormonal consideration is the *Drosophila* insulin-like peptide (Dilp), which acts in a similar fashion to vertebrate insulin by moderating cellular energy usage. Hugin and Dilp–secreting neurons thus further modulate responses to external chemosensory stimulation [90,114,119]. These neurons receive extensive input from both peripheral and internal sensory – but not external chemosensory – neurons, thus are included in the polysynaptic circuits that govern appropriate behavioural output selection based on both the peripheral sensory input and the internal state of the animal [90,119].

## Limitations of current approaches

Uncovering the processing logic of the brain has to-date been achieved mainly through connectomic analysis, coupled with genetic manipulation of animals at a single neuron level. Connectomics are useful in predicting the likely mechanisms of processing by understanding how neurons wire together. However, this analysis does not provide us with deeper, multidimensional information, chiefly the identity of the neurotransmitter or receptor type at each synapse. Furthermore, connectomic analyses are fundamentally limited by their resource-intensive nature, introducing the current problem of n = 1. This lack of reproducibility further jeopardizes one’s ability to confidently determine the wiring logic of the whole brain, and how much it differs between individuals, unless the scope is reduced to the level of subcircuits [128]. It is likely, however, that with the advent of novel computational approaches, such as use of artificial intelligence and machine learning, additional completed connectomes will be produced in a much quicker and easier manner, in addition to allowing for connectomic analysis of targeted mutants.

## Notable uncharacterized properties

Despite its numerical simplicity, many aspects of larval chemosensation, and in particular taste, remain unknown. For example, the identity and function of many peripheral neurons remains unclear. These include the basal neurons of the DOG, dorsolateral and distal neurons in the TOG, and six out of seven neurons in the VO (reviewed in [54]). Furthermore, the elusive odorant-binding proteins are yet to be discretely characterized in the larva, despite evidence of key functions in the adult including external signal modulation [129] and state-dependent transcriptional and behavioural changes [130,131].

A tantalizing missing piece in the larval taste story is the notable lack of a characterized mechanism for peripheral sugar sensation. The canonical sugar receptor, Gr43a, is only expressed in internal taste organs and the CNS and is definitively required for internal state-sensing of sugar [44]. Since CNS Gr43a neurons are by themselves sufficient for appropriate behavioural responses to sugar, it remains to be elucidated whether the ingestion of sugar is at play in this scenario. It is conceivable that peripheral sensation is required for ‘searching’ behaviour, while internal sensors convey the successful locating of sugar. Coupled with the fact that many GRs are yet to be de-orphanized, it is conceivable that sugar sensation will be uncovered in the peripheral organs through a more thorough interrogation of environments and chemosensory receptors. Furthermore, it is possible that sugar, a nutritional cue that is ubiquitous across the animal kingdom, is processed in a different manner to other tastants. For example, it is known that sugar signals from pharyngeal organs partially relay to the antennal lobe via octopaminergic neurons in the SEZ [83,109], suggesting some sugar-signal processing function in the olfactory system. Considering the pseudo-olfactory role of CO_2_-sensing gustatory receptors, it is possible that an opposing mechanism of taste-sensation may be facilitated by orphan ORs, such as Or2a, or broadly tuning promiscuous receptors, such as Or35a.
